# Management of an Active Oral Bleed Following Dental Implant Therapy Using Lidocaine and Epinephrine Injection in the Presence of an Oral Anticoagulant: A Clinical Report

**DOI:** 10.7759/cureus.29495

**Published:** 2022-09-23

**Authors:** Wesley D Banks, Yuli Breier, Peter Protzel, Michael Poulose

**Affiliations:** 1 Emergency Department, St. Joseph Medical Center, Bethpage, USA; 2 Anatomy Department, Touro College of Osteopathic Medicine, New York, USA; 3 Emergency Department, Staten Island University Hospital, New York, USA

**Keywords:** control of bleeding, oral and maxillofacial surgery, oral anticoagulants, emergency medicine, oral bleed, dental implantology, stop the bleed

## Abstract

Active bleeding in a patient on oral anticoagulants can be difficult to treat. While massive blood loss and hemorrhage are the highest concern, it is the incidental or seemingly benign bleeds that can eventually turn life-threatening. A deeper understanding of locating and controlling slow but constant minor bleeds that are resistant to clotting is important to consider and explore further.

## Introduction

Unstable bleeds are no stranger to the Emergency Department (ED). The struggle between Emergency Physicians and a bleeding patient is a tale seemingly as old as time. When we add blood thinners to the mix, however, we find ourselves in a much greater predicament. Blood thinning medications have multiple names, classes, and mechanisms of action. They also have a myriad of versatile uses, including, but not limited to, clot reduction, stroke prevention, myocardial infarction (MI) prevention, and so on. These medications, however, also have a major and highly particular adverse effect. This adverse effect is the inability to clot following an incidental bleed, preventing the blood from being able to coagulate properly which elevates accidental mortality. A good example comes from elderly patients on oral anticoagulants (OACs) for atrial fibrillation and/or flutter who have a greater risk for mortality compared with controls [1]. While the risk of a brain bleed after a slip and fall certainly outweighs that of a stroke or MI, there is a life-threatening element to oral anticoagulant medications unless we are able to successfully control bleeding in their presence. It is especially important for patients on blood thinners to be monitored for bleeding after undergoing surgery. In the case being presented, a 64-year-old male mentioned to his Oral and Maxillofacial Surgeon that he was taking an oral anticoagulant known as clopidogrel, but the surgeon did not discontinue the anticoagulant because he did not think it was necessary. It is very important for physicians to recognize how to stop the bleeding of their patients on anticoagulation medications, and this case offers a good example of a particular bleed that was difficult to control because the site of the bleed was particularly difficult to reach behind a fixed implant retained denture. When conventional methods such as compression, tranexamic acid, and Afrin failed to control the bleeding, it was a trial of injection lidocaine with epinephrine that ultimately stopped the bleeding.

## Case presentation

A 64-year-old male presented to the ED three days after having dental implants placed in the maxilla for a fixed hybrid denture prosthesis. The patient had mentioned to the Oral and Maxillofacial Surgeon that he was taking clopidogrel, but the physician decided discontinuation was unnecessary. In smaller procedures such as simple extractions, the continuation of OACs may be advisable. In this case, however, discontinuation should have been considered due to the more invasive nature of this particular procedure. The patient received a right-side dental implant-supported fixed denture, replacing all maxillary teeth. This requires multiple dental implants in the right and left maxilla for retention of the prosthesis. Upon arrival to the ED, the patient appeared disheveled, in a moderate amount of distress, and was wearing a blood-soaked t-shirt from an obvious oral bleed. The patient remained alert and oriented for the entirety of the visit. The patient stated that the bleed began approximately six hours before arrival, with the implant procedure being three days prior. No bleeding was noticed or appreciated until the initial onset six hours beforehand. On examination, the patient was bleeding from somewhere beneath his recently placed right-sided maxillary dental implants, but the exact source of the bleed could not be seen. His maxillary denture was fixed to the recently placed implants, and could not be removed to observe the bleeding site as they are meant to be permanent. The bleed was slow but constant, and suction was required every 10-15 minutes to maintain the patient’s airway efficiently due to pooling blood in the back of the oropharynx. International normalized ratio (INR) values, explained later, are used to measure blood coagulability. The patient had an INR of 1.3 on ED bloodwork which is considered mildly elevated. Although the discontinuation of OAC prior to surgery can be controversial depending on the procedure, both systematic review and retrospective analysis suggest that the absolute risk is low and there is no need to discontinue or alter the dose of the antithrombotic treatment for implant placement surgery safely [2,3]. It is still highly recommended to thoroughly screen patients for the medications they are on, but in this particular case, the discontinuation of clopidogrel may not have been advisable. Given that the location of the bleed was beneath the fixed denture and unreachable by the Emergency Physician, as opposed to an easily accessible tooth extraction site, for example, alternative measures to compression with topical clotting agents were needed when multiple local compression attempts had failed. The following sequence was used, in order, to try and resolve the bleeding following the failure of compression and topical clotting agent. Tranexamic acid (TXA) via soaked gauze was used. TXA is also a commonly used topical agent in cases of epistaxis, so utilizing the agent in other ENT situations would likely prove to be effective. In the case of this patient, however, the TXA-soaked gauze did not effectively control the bleeding. This is most likely once again attributed to the inability to reach the bleeding site beneath the prosthetic implant. The next attempt was aimed at using a mouth rinse of a brand-name nasal decongestant known as Afrin (oxymetazoline HCl). Traditionally, the medication is used for vasoconstriction of nasal blood vessels which will decrease mucous secretions. In this case, however, the vasoconstrictive properties would help sequester bleeding and potentially have a better chance of reaching the bleed being a liquid solution. Unfortunately, this was not effective in controlling the bleed, and additional measures were needed. By this point, Oral Surgery was consulted on the case by the Emergency Physician and the surgeon came in to evaluate the patient. Upon arrival, the surgeon requested local lidocaine 1% with epinephrine. While lidocaine is traditionally used for pain management during local procedures, the true purpose of this solution was for the epinephrine it contained. Epinephrine is a potent vasoconstrictor, and it happens to be injectable subcutaneously when necessary. The decision to inject areas of branching vessels surrounding the prosthetic was made to more effectively control the bleed further away from the actual site that remained invisible. The surgeon injected 4 mL of lidocaine with epinephrine (1:100,000) into the right hard and soft palate, as well as the buccal gingiva to gain full coverage around the prosthetic. After just a few minutes, the bleeding subsided. If the bleeding was unable to be controlled, the fixed dental prosthesis would have had to be removed and the site of the wound cauterized. This removal process is a technical dental procedure requiring special equipment and would take additional time to perform. The patient required intravenous fluids for volume replacement after losing approximately 1 L of blood while in the ED over the course of 2.5 hours. The patient was able to be discharged with a follow-up with his primary care provider in three to five days.

What ultimately makes this case important is the necessity to continue and expand our approach to treatments, while not being dismissive of seemingly benign cases. It is also to consider what order to use the clot-forming agents in, as well as considering the presence of OAC medications when traditional methods fail. It does not take much for a slow but constant bleed to lead to massive blood loss, and when we are unable to reach the site of a bleed, frontline compression or topical agents may not be viable options. The use of epinephrine was a great way to sequester bleeding by targeting surrounding areas and vasoconstricting the vessels that feed the likely source. A solid understanding of the pathophysiology behind a bleed while on blood thinners is important, especially in the ED. The order of use was also noteworthy as a clear stepwise approach may be highly beneficial, especially in the presence of blood-thinning agents such as clopidogrel. The presence of clopidogrel, in particular, makes this oral bleed important to understand, and a more useful case review.

## Discussion

**Figure 1:**
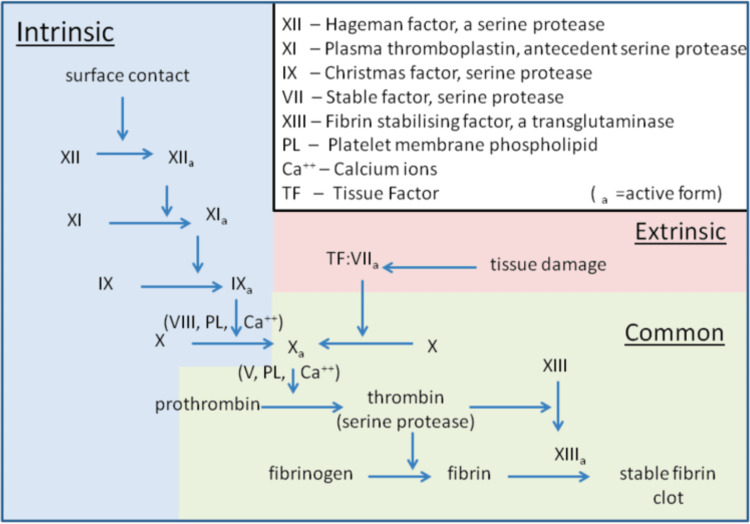
Intrinsic versus extrinsic pathway.

Treating patients who are actively bleeding and subsequently on OACs is not always an easy task. These medications, while useful in other facets of medicine, are not without their risk factors. Because they limit the body’s inherent ability to form blood clots, they elevate the possibility of a patient losing enough blood from a seemingly benign source that may result in serious harm or even death. A good example comes from elderly patients on OACs for atrial fibrillation and/or flutter in whom slips and falls have a greater mortality rate compared with controls [1]. Although there are many classes of drugs that alter the clotting process, to better understand the mechanism of the drugs, we must first discuss the process that occurs within the body. There are two major initiation pathways through which the body may perform hemostasis via blood clots. There is the intrinsic pathway, which is activated through exposure to endothelial collagen, and the extrinsic pathway, which is activated through tissue factor released by endothelial cells after external damage [4]. Both of these pathways eventually converge to the same specific point, known as the common pathway, leading to the activation of a clotting factor known as fibrin. Once activated, fibrin forms a mesh that ultimately stabilizes bleeding and forms a platelet plug. See Figure 1 for a more detailed outline of the factors involved, as well as the mechanism of action.

The mechanism by which coagulation allows for hemostasis is an intricate process that is done through a series of clotting factors. The intrinsic pathway consists of factors I, II, IX, X, XI, and XII. Respectively, each one is named, fibrinogen, prothrombin, Christmas factor, Stuart-Prower factor, plasma thromboplastin, and Hageman factor. The extrinsic pathway consists of factors I, II, VII, and X. Factor VII is called the stable factor. The common pathway consists of factors I, II, V, VIII, and X. These factors circulate through the bloodstream as zymogens and are activated into serine proteases. These serine proteases act as a catalyst to cleave the next zymogen into more serine proteases and ultimately activate fibrinogen. The following are serine proteases: factors II, VII, IX, X, XI, and XII. The following are not serine proteases: factors V, VIII, and XIII. The intrinsic pathway is activated through exposed endothelial collagen, and the extrinsic pathway is activated through tissue factor released by endothelial cells after external damage [4].

**Figure 2:**
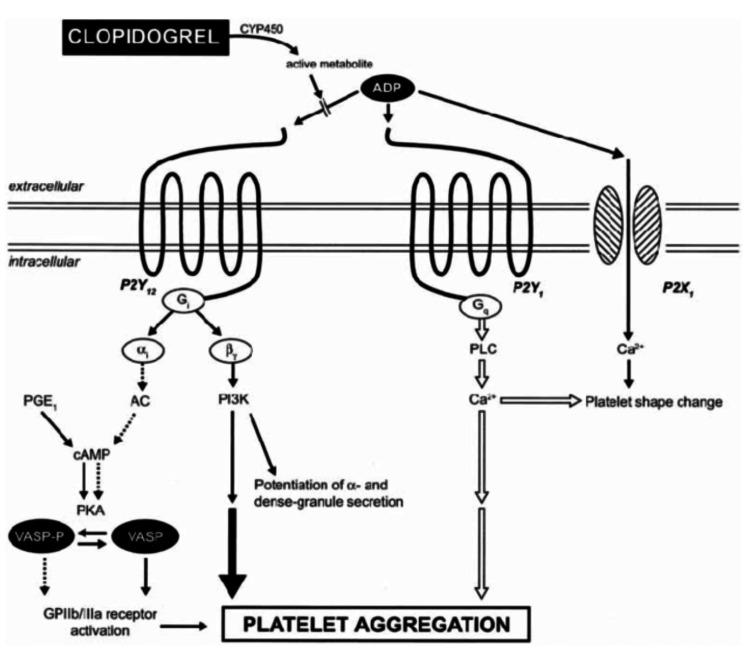
Mechanism of action of clopidogrel. Multiple arrows indicate that intermediate steps may be involved; dotted arrows = inhibition; solid arrows = activation. CYP450 = cytochrome P450; PGE 1 = prostaglandin E 1; PKA = protein kinase activation; PLC = phospholipase C

The method of controlling a bleed such as local oral bleeds is referred to as local hemostatic measures. These measures traditionally include local compression with absorbable gelatin powder, TXA, and Afrin (oxymetazoline HCL). Now that we have a better understanding of the clotting process, we can begin to analyze the mechanism of OACs, with a specific focus on Plavix (clopidogrel). Clopidogrel is a pro-drug in which the active metabolite irreversibly inhibits the adenosine diphosphate (ADP) P2Y 12 receptor. The full mechanism of action of clopidogrel can be seen in Figure 2.

Clopidogrel is a pro-drug that is metabolized by the cytochrome P450 system to generate the active metabolite which irreversibly inhibits the ADP P2Y 12 receptor. ADP binds to the P2Y 1 and P2X 1 receptors leading to a change in platelet shape initiating a weak and transient phase of platelet aggregation. The binding of ADP to its G i-coupled P2Y 12 receptor liberates the G i protein subunits α Gi and βγ, which results in the stabilization of platelet aggregation. The subunit α Gi leads to the inhibition of adenylyl cyclase (AC), which reduces cyclic adenosine monophosphate (cAMP) levels. This inhibits the cAMP-mediated phosphorylation of vasodilator-stimulated phosphoprotein (VASP) (VASP-P), which is known to be closely related to the inhibition of glycoprotein IIb/IIIa receptor activation [5].

Coagulability can be tracked using routine blood work analysis such as prothrombin time (PT), partial thromboplastin time (PTT), or INR. PT traditionally reflects the extrinsic pathway’s functionality, while PTT/INR represents the intrinsic pathway [6]. In any situation, an elevation in these values demonstrates a longer period of time required for clot formation. In general, an INR of 1.1 or less is considered normal, and sufficient clotting can be expected. An INR in the range of 2.0-3.0 is widely considered to be the ideal therapeutic range of a patient on a standard OAC such as warfarin [7] (a competitive inhibitor of VKORC1). The first and most effective choice in local hemostasis management of oral bleeding is compression with the application of a topical hemostatic agent to the site (such as absorbable gelatin powder), as well as the placement of sutures [8]. In the case of oral bleeds, tooth extraction is one of the most common sources of such a bleed, and compression is often sufficient even among patients with INR values of up to 5.6 [9]. TXA is a synthetic reversible competitive inhibitor to the lysine receptor found on plasminogen. The binding of this receptor prevents plasmin (activated form of plasminogen) from binding to and ultimately stabilizing the fibrin matrix [10]. Afrin is a direct-acting sympathomimetic that stimulates adrenergic receptors, causing vasoconstriction of dilated arterioles and reducing blood flow [11].

## Conclusions

The purpose of this case study is to provide an in-depth look at the steps taken, in order, during a case of an oral bleed in the presence of OACs such as clopidogrel. In many seemingly benign cases that present to the ED routinely, some, if not all, of these incidents can lead to what is ultimately a life-threatening predicament. It is important to have measures in place for when the traditional interventions of a slow but prolonged bleed take place over a long period of time. While the issue may be seemingly easy to correct with simple compression of the wound site, we may not always be able to reach the site of the bleed in cases of the presence of a fixed dental prosthesis, trauma, or other physical obstructions. The stepwise approach to treatment and understanding of pathophysiology can be lifesaving in these types of cases.
